# Phenotyping Dysautonomia in Unexplained Syncope: Diagnostic Yield and Therapeutic Implications in a Moroccan Cohort

**DOI:** 10.7759/cureus.101268

**Published:** 2026-01-10

**Authors:** Oumaima Taoussi, Hibat Allah Kamri, Soukaina Scadi, Benouna Ghali, Fatimazahra Merzouk

**Affiliations:** 1 Cardiology, Mohammed VI International University Hospital, Mohammed VI University of Health Sciences (UM6SS), Casablanca, MAR; 2 Cardiology, Cheikh Khalifa International University Hospital, Mohammed VI University of Health Sciences (UM6SS), Casablanca, MAR

**Keywords:** autonomic nervous system testing, dysautonomia, orthostatic hypotension, postural orthostatic tachycardia syndrome, unexplained syncope

## Abstract

Background and objective

Unexplained syncope remains a frequent and challenging clinical problem, even after guideline-directed cardiovascular and neurological evaluation. In a substantial proportion of patients, syncope remains without an identified etiology after conventional investigations, suggesting alternative underlying mechanisms. Autonomic nervous system (ANS) dysfunction plays a central role in cardiovascular regulation and has been increasingly recognized as a key contributor to unexplained syncope.

ANS testing allows objective evaluation of cardiovascular autonomic reflexes, including sympathetic and parasympathetic responses to orthostatic and physiological stressors. The objective of this study was to assess autonomic profiles using standardized ANS testing in patients referred for unexplained syncope and to characterize dysautonomic patterns that may contribute to syncope mechanisms.

Methods

We conducted a retrospective mono-centric study including 90 consecutive adult patients referred to a tertiary autonomic unit for syncope remaining without an identified etiology after conventional evaluation between January 2024 and June 2025. All patients underwent a standardized ANS testing battery, including an active standing (orthostatic) test, a deep-breathing test, an isometric handgrip test, and a mental stress test. Demographic data, cardiovascular risk factors, clinical presentation, and ANS test results were systematically collected and analyzed.

Results

ANS testing identified autonomic abnormalities considered clinically relevant in a high proportion of patients with unexplained syncope despite routine conventional investigations. The most frequent findings included exaggerated vagal responses, orthostatic intolerance syndromes such as postural orthostatic tachycardia syndrome, sympathetic alpha-adrenergic dysfunction leading to orthostatic hypotension, and mixed autonomic profiles. Overlapping autonomic phenotypes were frequently observed within the same patient, underscoring the complexity of dysautonomic syncope.

Conclusion

ANS testing provides a high diagnostic yield in patients with syncope remaining without an identified etiology after standard evaluation by revealing clinically relevant autonomic dysregulation. Integration of ANS testing as a second-line diagnostic approach may improve etiological classification and support individualized management strategies in selected patients.

## Introduction

Syncope is defined as a transient loss of consciousness caused by global cerebral hypoperfusion, characterized by rapid onset, short duration, and spontaneous complete recovery. It represents a common reason for emergency department visits and specialist referrals, imposing a substantial clinical and socioeconomic burden due to recurrence, trauma-related injuries, and impaired quality of life. Despite structured diagnostic pathways, approximately 20-40% of patients remain without a clearly identified etiology after standard evaluation [1]. In this context, syncope is considered unexplained when it remains without an identified cause after guideline-directed assessment, typically including detailed history taking, physical examination with orthostatic vital signs, baseline electrocardiography, and appropriate cardiovascular and neurological investigations based on clinical suspicion [1,2].

Current guidelines from the 2018 European Society of Cardiology (ESC) and the 2017 ACC/AHA/HRS emphasize that, following exclusion of high-risk cardiac causes, reflex syncope and orthostatic intolerance syndromes account for a large proportion of cases [1,2]. However, diagnostic uncertainty frequently persists, particularly when syncopal events are intermittent, situational, or poorly documented. Conventional cardiovascular and neurological investigations are primarily designed to identify structural disease, arrhythmias, or epileptic disorders, but they often fail to capture dynamic abnormalities of cardiovascular regulation. In this context, dysfunction of the autonomic nervous system (ANS) has emerged as a key pathophysiological mechanism, given its central role in regulating heart rate, vascular tone, and blood pressure responses to postural changes and physiological stress.

ANS testing provides an objective assessment of cardiovascular autonomic reflexes by evaluating both parasympathetic (cardiovagal) and sympathetic (adrenergic) components through standardized provocative maneuvers and orthostatic challenges. Consensus statements support the use of tilt testing and complementary cardiovascular autonomic tests, such as active standing, deep breathing, and Valsalva-based protocols, when the initial clinical evaluation suggests reflex syncope, orthostatic hypotension (including delayed forms), postural orthostatic tachycardia syndrome (POTS), or psychogenic pseudosyncope but remains inconclusive [3]. In routine clinical practice, non-tilt ANS tests are often underutilized despite their accessibility, physiological relevance, and ability to capture subtle autonomic abnormalities under real-world conditions.

Among orthostatic intolerance syndromes, POTS is characterized by an excessive increase in heart rate upon standing in the absence of significant orthostatic hypotension and is associated with heterogeneous clinical manifestations and underlying mechanisms. Expert consensus highlights the importance of standardized diagnostic criteria and structured evaluation, including ANS testing when appropriate [4]. Similarly, orthostatic hypotension, particularly neurogenic forms, requires careful phenotypic characterization due to its distinct prognostic and therapeutic implications and impact on patient management [5,6]. Importantly, autonomic disorders frequently coexist, and mixed or overlapping autonomic phenotypes may better reflect the complexity of syncope mechanisms than single-mechanism classifications.

Despite these considerations, real-world data describing comprehensive ANS testing profiles in consecutive patients referred for syncope remaining without an identified etiology after standard evaluation remain limited. This gap is particularly evident in North African clinical settings, where specialized autonomic units are scarce and regional patterns of referral, comorbidities, and healthcare access may differ from those reported in European or North American cohorts. Therefore, this study aimed to characterize autonomic phenotypes using a standardized battery of ANS tests in a cohort of patients evaluated for unexplained syncope and to identify dysautonomic patterns that may improve mechanistic understanding and support individualized management strategies.

## Materials and methods

Study design and population

This retrospective, single-center cohort study was conducted at the Autonomic Nervous System Exploration Unit of the Mohammed VI University Hospital. We included 90 consecutive adult patients (aged ≥18 years, with no upper age limit) referred for the evaluation of syncope remaining without an identified etiology between January 2024 and June 2025. Syncope was defined as a transient loss of consciousness with rapid onset, spontaneous complete recovery, and no residual neurological deficit.

All patients had syncope that remained without an identified etiology after a standardized, guideline-directed initial evaluation, including a detailed medical history, physical examination with orthostatic vital signs, and a 12-lead electrocardiogram. All patients had normal transthoracic echocardiography and 24-hour Holter ECG monitoring. Additional complementary investigations, such as exercise testing, electroencephalography, or brain imaging, were performed when clinically indicated and were non-diagnostic. Tilt-table testing had not been performed before referral to the autonomic unit.

Patients with identified cardiac, neurological, metabolic, or psychogenic causes of syncope, severe psychiatric or cognitive disorders, or incomplete autonomic testing were excluded. “Consecutive” referrals included all eligible patients referred during the study period who completed the full standardized autonomic testing protocol.

Ethical considerations

The study used fully anonymized data collected during routine clinical care. In accordance with local regulations, formal approval from an Institutional Review Board was not required. Written informed consent for the use of clinical data for research purposes was obtained from all participants.

ANS testing protocol

Adult patients were defined as individuals aged ≥18 years, with no upper age limit applied. Consecutive referrals included all eligible patients referred during the study period who completed the full standardized ANS testing battery.

Unexplained syncope was operationally defined as syncope remaining without an identified etiology after guideline-directed conventional evaluation, including clinical assessment, electrocardiography, and at least one normal complementary cardiovascular or neurological investigation; tilt-table testing had not been systematically performed prior to referral.

Medications known to influence autonomic function were temporarily withheld before testing when clinically safe. All autonomic tests were conducted according to international consensus protocols, using standardized durations, fixed testing sequences, and established heart rate and blood pressure thresholds. Beat-to-beat heart rate and blood pressure were recorded using validated non-invasive monitoring systems.

Test interpretation was performed by experienced clinicians using predefined diagnostic criteria. Patients with overlapping abnormalities were classified as having a conclusive dysautonomic profile when at least one abnormality provided a plausible mechanism for syncope. Symptom reproduction during testing was recorded when present and considered supportive but not mandatory for classification.

Missing data were minimal and handled using complete-case analysis. Given the exploratory nature of the study, no adjustment for multiple comparisons was applied.

Data collection and definition of autonomic profiles

Demographic characteristics, clinical history (including syncope characteristics), and cardiovascular risk factors (physician-diagnosed) were extracted from electronic medical records. Based on an integrated interpretation of autonomic test results, patients were classified as having either a conclusive dysautonomic profile, when at least one abnormal finding provided a physiologically plausible autonomic mechanism for syncope, or a non-conclusive autonomic profile when testing was normal or non-diagnostic.

When multiple autonomic abnormalities were present, they were considered coexisting mechanisms rather than mutually exclusive categories.

Statistical analysis

Statistical analysis was performed using IBM SPSS Statistics for Windows, Version 25 (Released 2017; IBM Corp., Armonk, New York, United States). Continuous variables are presented as mean ± standard deviation or median with interquartile range, as appropriate, while categorical variables are expressed as numbers and percentages. Comparisons between groups were conducted using Student’s t-test or the Mann-Whitney U test for continuous variables and the chi-square test or Fisher’s exact test for categorical variables. Analyses were performed using complete-case data, as missing values were minimal. Given the exploratory nature of the study, no correction for multiple comparisons was applied. A two-tailed p-value below 0.05 was considered statistically significant.

## Results

Study population and clinical presentation

A total of 90 adult patients (≥18 years) referred for the evaluation of syncope remaining without an identified etiology after guideline-directed conventional assessment were included. The mean age was 60.0 ± 18.5 years (range: 18-87), with a female predominance (64.4%). Baseline demographic characteristics and physician-diagnosed cardiovascular risk factors are summarized in Table 1.

**Table 1:** Baseline characteristics of the study population SD: Standard deviation. Values are expressed as mean ± standard deviation or number (percentage). Cardiovascular risk factors include hypertension, diabetes mellitus, dyslipidemia, and active smoking.

Characteristic	Value
Age	
Range (years)	18 – 87
Mean ± SD (years)	60.0 ± 18.5
Sex, n (%)	
Female	58 (64.4)
Male	32 (35.6)
Cardiovascular Risk Factors, n (%)	
Hypertension	9 (10.0)
Diabetes Mellitus	4 (4.4)
Dyslipidemia	4 (4.4)
Active Smoking	11 (12.2)
Menopausal Status (Women only), n/N (%)	
Postmenopausal	26/58 (44.8)

Syncope occurred as a single episode in 59 patients (65.6%), while 31 patients (34.4%) experienced recurrent syncope (≥2 episodes). Prodromal symptoms were frequently reported and included dizziness, visual dimming, nausea, and diaphoresis. Syncopal events most commonly occurred in the upright position or during postural change, and were often triggered by prolonged standing, emotional stress, or situational factors. Exertional syncope was uncommon.

The interval between the last syncopal event and autonomic testing varied according to referral logistics, generally ranging from several days to a few weeks.

Conventional paraclinical investigations

All patients underwent a standardized initial evaluation including clinical assessment, orthostatic vital signs, and 12-lead electrocardiography, which did not identify a causal etiology. Transthoracic echocardiography was systematically performed and was normal in all cases.

Additional investigations were obtained based on clinical suspicion to minimize indication bias. Twenty-four-hour Holter monitoring was performed in 42 patients (46.7%), exercise stress testing in 12 patients (13.3%), and coronary angiography in four patients (4.4%), all without diagnostic findings. Neurological investigations, including brain imaging and electroencephalography, were performed when clinically indicated and were normal. Tilt-table testing had not been routinely performed prior to referral, as patients were referred specifically for comprehensive autonomic evaluation.

Orthostatic testing results

Orthostatic testing revealed heterogeneous cardiovascular responses. Orthostatic hypotension was observed in 26 patients (28.9%). Orthostatic tachycardia not fulfilling criteria for POTS was identified in 18 patients (20.0%). POTS, defined by a sustained heart rate increase ≥30 beats/min within 10 minutes of standing in the absence of orthostatic hypotension, was diagnosed in 14 patients (15.6%), including two patients (2.2%) classified as complicated POTS due to associated blood pressure instability and marked symptom burden.

Orthostatic hypertension was observed in 10 patients (11.1%), and a Bezold-Jarisch reflex pattern in six patients (6.7%). A normal orthostatic response was observed in 14 patients (15.6%). Percentages were not mutually exclusive, as overlap between orthostatic phenotypes occurred in some patients, reflecting coexisting mechanisms rather than exclusive categories. Symptom reproduction during orthostatic testing was recorded when present and was supportive, but not mandatory, for phenotype classification.

Parasympathetic (cardio-vagal) function

Cardio-vagal assessment using the deep breathing test demonstrated vagal hyperactivity in 68 patients (75.6%), normal responses in 12 patients (13.3%), and vagal deficiency in 10 patients (11.1%). Age-adjusted reference values were applied.

During isometric handgrip testing, vagal hyperactivity was observed in 58 patients (64.4%), vagal deficiency in 18 patients (20.0%), and normal responses in 14 patients (15.6%). In cases of discordant findings between tests, interpretation was based on integrated assessment rather than isolated parameters.

Sympathetic function

Mental stress testing revealed beta-adrenergic hyperactivity in 63 patients (70.0%), deficiency in 22 patients (24.4%), and normal responses in five patients (5.6%). Central alpha-adrenergic responses showed hyperactivity in 62 patients (68.9%) and deficiency in 24 patients (26.7%). Peripheral alpha-adrenergic testing demonstrated hyperactivity in 76 patients (84.4%) and deficiency in 10 patients (11.1%). Central and peripheral sympathetic abnormalities were analyzed separately and interpreted jointly within the global autonomic profile.

Medications influencing autonomic function were temporarily withheld when clinically safe, and mental stress testing was conducted under standardized conditions to minimize anxiety-related effects.

A comprehensive summary of autonomic testing results is presented in Table 2.

**Table 2:** Summary of autonomic nervous system testing results Values are expressed as number (percentage). POTS: postural orthostatic tachycardia syndrome.

Autonomic domain and testing		Response category	n (%)
Orthostatic function	Orthostatic test	Orthostatic hypotension	26 (28.9)
		Orthostatic tachycardia (non-POTS)	18 (20.0)
		Normal response	14 (15.6)
		POTS	14 (15.6)
		Complicated POTS	2 (2.2)
		Orthostatic hypertension	10 (11.1)
		Bezold–Jarisch reflex	6 (6.7)
Parasympathetic function	Deep breathing test	Vagal hyperactivity	68 (75.6)
		Normal response	12 (13.3)
		Vagal deficiency	10 (11.1)
	Handgrip test	Vagal hyperactivity	58 (64.4)
		Normal response	14 (15.6)
		Vagal deficiency	18 (20.0)
Sympathetic function	Mental stress test	Beta-sympathetic hyperactivity	63 (70.0)
		Normal response	5 (5.6)
		Beta-sympathetic deficiency	22 (24.4)
	Central alpha response	Alpha-sympathetic hyperactivity	62 (68.9)
		Normal response	4 (4.4)
		Alpha-sympathetic deficiency	24 (26.7)
	Peripheral alpha response	Hyperactivity	76 (84.4)
		Normal response	4 (4.4)
		Deficiency	10 (11.1)

Integrated autonomic profiles

Based on integrated interpretation of all autonomic domains, 76 patients (84.4%) were classified as having a conclusive dysautonomic profile, defined by the presence of at least one autonomic abnormality considered physiologically sufficient to explain syncope. Fourteen patients (15.6%) exhibited a non-conclusive profile, characterized by normal or non-diagnostic findings. Mixed or overlapping autonomic abnormalities were common within the conclusive group and were classified pragmatically without hierarchical weighting.

Comparative analysis between autonomic profiles

Comparative analysis between patients with conclusive (n = 76) and non-conclusive (n = 14) autonomic profiles is summarized in Table 3.

**Table 3:** Comparative analysis between conclusive and non-conclusive autonomic profiles SD: Standard deviation; CV: Cardiovascular. Values are expressed as mean ± standard deviation or number (percentage). Continuous variables were compared using the Mann–Whitney U test. Categorical variables were compared using the chi-square test or Fisher’s exact test, as appropriate. A p-value < 0.05 was considered statistically significant.

Variable	Overall (N=90)	Conclusive dysautonomia (n=76)	Non-conclusive profile (n=14)	p-value	Test statistic
Age, years (mean ± SD)	60.0 ± 18.5	56.2 ± 17.1	65.8 ± 12.3	0.045	U = 332.0
Female sex, n (%)	58 (64.4)	48 (63.2)	10 (71.4)	0.552	χ² = 0.35
Recurrent syncope (≥2 episodes), n (%)	30 (33.3)	28 (36.8)	2 (14.3)	0.093	χ² = 2.85
Cardiovascular Risk Factors, n (%)					
Hypertension	9 (10.0)	6 (7.9)	3 (21.4)	0.142	Fisher's exact test
Diabetes Mellitus	4 (4.4)	2 (2.6)	2 (14.3)	0.112	Fisher's exact test
Dyslipidemia	4 (4.4)	3 (3.9)	1 (7.1)	0.516	Fisher's exact test
Active smoking, n (%)	11 (12.2)	9 (11.8)	2 (14.3)	0.678	χ² = 0.07
Any CV Risk Factor Present, n (%)	25 (27.8)	18 (23.7)	7 (50.0)	0.043	χ² = 4.12

Patients with a conclusive dysautonomic profile were significantly younger than those with a non-conclusive profile (56.2 ± 17.1 vs. 65.8 ± 12.3 years, p = 0.045). This comparison was performed using the Mann-Whitney U test due to non-normal age distribution on preliminary assessment, although values are reported as mean ± standard deviation for descriptive purposes. No multivariable adjustment for potential confounders such as sex or cardiovascular risk factors was performed, given the exploratory design and the limited size of the non-conclusive group.

Sex distribution did not differ significantly between groups (female sex: 63.2% vs. 71.4%, p = 0.552). Recurrent syncope was analyzed as a binary variable (single episode vs. ≥2 episodes). It was more frequent in the conclusive group (36.8% vs. 14.3%), although this difference did not reach statistical significance (p = 0.093). No alternative ordinal or multivariable modeling was explored due to sample size limitations.

Individual cardiovascular risk factors were numerically more prevalent in the non-conclusive group. Cardiovascular risk burden was analyzed using a composite endpoint in which risk factors were weighted equally; clustering or multivariable modeling of individual risk factors was not performed. The presence of at least one cardiovascular risk factor was significantly more frequent among patients with a non-conclusive profile (50.0% vs. 23.7%, p = 0.043).

Effect sizes and confidence intervals were not systematically reported, as the primary objective of the study was descriptive phenotyping rather than inferential risk modeling. Given the small size of the non-conclusive group, non-significant findings should be interpreted cautiously due to limited statistical power.

Stratified analyses by age or sex and correction for multiple comparisons were not performed, in keeping with the exploratory nature of the study.

Overall, patients with conclusive dysautonomic profiles tended to be younger and exhibited a lower burden of traditional cardiovascular risk factors.

## Discussion

This study of 90 consecutive patients referred for unexplained syncope demonstrates that comprehensive ANS evaluation identifies a conclusive dysautonomic profile in the majority of cases (84.4%). Importantly, this diagnostic yield refers to autonomic abnormalities judged clinically relevant and physiologically plausible to explain syncope, rather than to the detection of any minor or isolated autonomic deviation. These findings highlight autonomic dysregulation as a predominant mechanism when standard cardiovascular and neurological investigations are unrevealing and provide original data from a North African population that remains underrepresented in the literature [1,2]. The high diagnostic yield should nevertheless be interpreted within the context of a tertiary autonomic referral center, where patients are highly selected after extensive conventional evaluation, potentially enriching the cohort in dysautonomic conditions compared with unselected syncope populations.

The predominance of vagal hyperactivity, observed in 75.6% of patients, supports the central role of exaggerated parasympathetic activation in syncope pathophysiology. Laboratory-defined vagal hyperactivity reflects an increased susceptibility of cardioinhibitory and vasodepressor reflexes rather than a direct one-to-one correlation with spontaneous syncopal events, which may remain intermittent and situational [7,8]. In parallel, orthostatic hypotension (28.9%) and POTS (17.8%) were frequently identified, emphasizing impaired sympathetic cardiovascular adaptation to orthostasis [9,10]. The overlap observed between vagal hyperactivity, orthostatic hypotension, and POTS likely reflects coexisting autonomic mechanisms within the same patient rather than testing-related artifacts, illustrating the complexity of real-world dysautonomia. Taken together, these findings confirm that orthostatic intolerance syndromes represent a major cause of syncope once structural cardiac disease has been excluded [1,11].

Beyond parasympathetic predominance, experimental and clinical data indicate that abnormalities in sympathetic nerve activity also contribute to the hemodynamic cascade leading to fainting, particularly during orthostatic stress and reflex-mediated syncope [12]. The identification of specific reflex patterns, including those consistent with the Bezold-Jarisch reflex, further illustrates the diagnostic value of provocative ANS testing in uncovering complex autonomic mechanisms that are often missed during routine clinical evaluation [8].

Comparative analysis revealed distinct patient profiles. Individuals with a conclusive dysautonomic profile were younger and exhibited fewer traditional cardiovascular risk factors, a phenotype typically described in primary dysautonomias such as vasovagal syncope and POTS [10,13]. Age-related physiological changes in autonomic regulation may partly explain the higher frequency of non-conclusive profiles among older patients, in whom mixed cardiovascular, pharmacological, or degenerative mechanisms may coexist. Conversely, patients with non-conclusive autonomic testing were older and more frequently presented with cardiovascular risk factors, suggesting alternative or multifactorial mechanisms, including intermittent arrhythmias, conduction abnormalities, or medication-related effects that are not systematically detected by standard ANS testing protocols [14,15]. These observations reinforce the heterogeneous nature of unexplained syncope and underline the importance of individualized diagnostic strategies.

From a clinical perspective, these findings support the role of ANS testing as a second-line investigation within guideline-based diagnostic pathways, after exclusion of high-risk cardiac causes and when initial evaluation remains inconclusive [2,3]. Identifying a specific autonomic phenotype enables management tailored to the underlying mechanism, ranging from non-pharmacological measures for vagal syncope to targeted therapeutic approaches for POTS or neurogenic orthostatic hypotension [16-18]. The identification of mixed or complex autonomic phenotypes is particularly relevant, as it encourages personalized counseling and combined management strategies rather than oversimplified single-mechanism labeling, with potential implications for prognosis and treatment stratification [19,20].

Short-term follow-up over three months suggested symptomatic improvement in the majority of patients after diagnostic clarification and implementation of lifestyle and hygieno-dietetic measures. This improvement was based on routine clinical reassessment and patient-reported outcomes, without standardized quantitative scales. These observations highlight the therapeutic value of diagnostic clarification, patient education, and reassurance, but should be interpreted cautiously, as the absence of a control group precludes causal inference regarding management impact.

Several limitations should be acknowledged. The retrospective, single-center design may limit generalizability, and referral bias inherent to a tertiary autonomic unit may overestimate the prevalence of dysautonomia. Autonomic response classification relied on laboratory-specific normative values, although these were consistent with international consensus recommendations [9]. Temporary withdrawal of medications influencing autonomic function prior to testing may also have affected autonomic responses and diagnostic classification. The relatively small number of patients with non-conclusive testing limited statistical power for certain comparisons, and the short follow-up duration precluded conclusions regarding long-term outcomes or treatment efficacy.

Future studies should adopt prospective, multicenter designs to validate these findings across broader populations and healthcare settings [21]. Longer follow-up periods, standardized outcome measures, and incorporation of advanced autonomic assessment techniques, such as heart rate variability analysis or sudomotor testing, may allow more refined phenotyping and improved prognostic stratification [21]. Finally, data from North African cohorts such as ours contribute to a more global understanding of dysautonomia by accounting for regional differences in referral patterns, comorbidities, and healthcare access, and may help inform future updates of clinical algorithms for unexplained syncope [22].

## Conclusions

In conclusion, comprehensive ANS evaluation is a high-yield and clinically meaningful step in the diagnostic workup of patients with syncope that remains without an identified etiology after conventional assessment. By uncovering underlying dysautonomic mechanisms, ANS testing moves the diagnostic process beyond exclusion toward positive phenotyping, thereby enabling clinicians to understand the physiological substrate of syncope better. The identification of a conclusive autonomic profile not only refines etiological classification but also facilitates individualized, mechanism-oriented management strategies. Importantly, our findings suggest that diagnostic clarification, combined with targeted non-pharmacological measures, may alleviate symptom burden and improve patient confidence, even in the absence of complex therapeutic interventions. Integrating structured ANS testing into syncope care pathways may therefore enhance diagnostic efficiency, personalize management, and ultimately improve the quality of care for patients with unexplained or recurrent syncope.
